# Acknowledgement to Reviewers of *Cancers* in 2017

**DOI:** 10.3390/cancers10010015

**Published:** 2018-01-10

**Authors:** Cancers Editorial Office

**Affiliations:** MDPI AG, St. Alban-Anlage 66, 4052 Basel, Switzerland; cancers@mdpi.com

Peer review is an essential part in the publication process, ensuring that Cancers maintains high quality standards for its published papers. In 2017, a total of 172 papers were published in the journal. Thanks to the cooperation of our reviewers, the median time to first decision was 21 days and the median time to publication was 44 days. The editors would like to express their sincere gratitude to the following reviewers for their time and dedication in 2017:
Advani, SunilLin, HaiboAggarwal, Bharat B.Lin, Huey-JenAlbano, EmanueleLindqvist, LisaAlevizopoulos, KonstantinosLiu, BotaoAlkan, SerhanLiu, Jun-JenAllen, ChristopherLiu, DelongAmato, RosarioLiu, YongqingAmato, Robert J.Liu, Yen-NienAmrutkar, ManojLocati, Laura D.An, SaiLuftig, MicahAndersen, Jens RikardtLumniczky, KatalinAnsa, BenjaminLundholm, Marie LundholmAntonescu, CostinLundstrom, KennethArfuso, FrankLuo, Yong-ZhangArgani, PedramLuscinskas, Francis W.Arora, Vivek K.Ma, PatrickAsiedu, MichaelMace, ThomasAtherly, AdamMaffucci, TaniaAveic, SanjaMaggiolini, MarcelloAvila, Matias A.Mahimainathan, LeninAzmi, Asfar S.Månsson, ChristopherBabib, AmynMaréchal, AlexandreBagnasco, AnnamariaMarimpietri, DaniloBai, ShuhuaMartelli, AlbertoBaniahmad, AriaMartin, Sally K.Bansal, AjayMartinon, FabioBarbagallo, DavideMasuelli, LauraBarberi-Heyob, Muriel A.Matsugaki, AiraBarbolina, Maria V.Matsui, JunjiBasu, AlakanandaMaugeri, MarcoBattistelli, CeciliaMawhinney, ThomasBattistini, LuciaMcClelland, SarahBayascas, JoseMcKeague, MaureenBeaman, Kenneth D.Mckillop, Iain H.Becker, Therese M.Medin, Jeffrey A.Beebe, Stephen J.Medová, MichaelaBeierle, ElizabethMehta, ShwetalBeltran, ChrisMeijerink, Martijn R.Benistant, ChristineMendenhall, NancyBenjamin, DonMenon, SmithaBentel, Jacqueline M.Miller, Jeffrey S.Bergamo, AlbertaMiller, Alexandra C.Bergh, AndersMiyoshi, EijiBergmann, AndreasMo, Yin-YuanBigey, PascalModjtahedi, HelmoutBinder, MaschaMorales, JulioBizzarri, MarianoMorioka, ShoBlack, Esther P.Mudryj, MariaBlons, HélèneMuñoz, Aránzazu SánchezBoddicker, Rebecca L.Murray, Paul G.Bodnar, LubomirMuto, ManabuBoehme, KarlNadiminty, NagalakshmiBonzheim, IrinaNagaraju, Ganji PurnachandraBooken, NinaNagel, ZacharyBora-Singhal, NamrataNakamura, AsakoBordonaro, MichaelNakaseko, ChiakiBorrebaeck, Carl A.K.Nandi, SaikatBoström, Jan PatrickNarita, NorihikoBouffler, SimonNatarajan, Sathish KumarBourgeois-Daigneault, Marie-ClaudeNazarian, JavadBournet, BarbaraNeureiter, DanielBown, StephenNevzorova, Yulia A.Brandao, JoaoNi, XiaoBreckpot, KarineNishimura, NoriyukiBreitenbücher, FrankNock, NoraBuchholz, JuliaNowsheen, SomairaBurman, PiaO’reilly, Eileen M.Calin, GeorgeOgino, ShujiCalvisi, Diego F.Oosting, Sjoukje F.Cao, BoOrdónez-Mena, José M.Carosella, Edgardo D.Oschlies, IlskeCarpinteiro, AlexanderOve, RogerCarter, DavidPagel, MartyCarver, Brett S.Paggetti, JérômeCerchia, LauraPalmer, Ruth H.Ceresa, BrianPalmirotta, RaffaeleChalmers, Jeffrey J.Parrington, JohnChandrasiri, UpekshaPastor, FernandoChantrill, Lorraine A.Pedraza-Chaverri, JoséChatterjee, JhinukPellat-Deceunynck, CatherineChen, Zhuo (Georgia)Peña, Maria Marjorette O.Chen, Kun-MingPinzani, PamelaChen, ZhongPlymate, Stephen R.Chevret, EdithPocheć, EwaChoi, Yoon-LaPonz-Sarvisé, MarianoChou, Cheng-YangPorta, CamilloChoudhury, MalayPosey, Avery D.Choulier, LaurencePotiron, VincentChristensen, James G.Primo, LucaChung, Eun JooPulliero, AlessandraCicenas, JonasPyka, ThomasClark, GeoffreyQian, HongClere, NicolasQuintanilla-Martinez, LeticiaColey, Helen M.Rahib, LolaColler, HilaryRaimondo, StefaniaColson, NatalieRainero, ElenaCooke, MarcusRamakrishnan, SadeeshCoppedè, FabioRassidakis, George Z.Corn, PaulReales-Calderón, Jose AntonioCortese, KatiaRedmer, TorbenCrnogorac-Jurcevic, TatjanaRedondo Muñoz, JavierCurtis, SarahRenaudineau, YvesDal Corso, AlbertoRepasky, Elizabeth A.Dalby, Kevin N.Ricci, GiuliaDamia, GiovannaRich, Jeremy N.Davies, MichaellRizvi, ImranDavis, RichardRobles, AnaDe, PradipRödel, FranzDegrassi, FrancescaRodriguez-Lafrasse, ClaireDevys, DidierRomani, AndreaDeWees, ToddRooj, ArunDey, NandiniRooman, IlseDharmarajan, ArunRoos, Wynand P.Di Blasio, LauraRosell, RafaelDi Fazio, PietroRosenzweig, AllisonDormond, OlivierRübe, Claudia E.Dovat, SinisaRussell, RyanDurham, Andrew L.Sabatini, Linda M.Duvvuri, UmamaheswarSaha, AchintoDy, GraceSaini, ShikhaEid, NabilSaini, SharanjotEl-Heliebi, AminSainz Jr., BrunoEngelberg, DavidSalaspuro, MikkoFaber, Anthony C.Saltman, David L.Feng, HuiSamuel, TemesgenFeng, Liu-SmithSánchez-Tilló, EsterFerretti, ElisabettaSantoni-Rugiu, EricFilleur, StephanieSarkar, SaswataFitzgerald, Rebecca C.Scaltriti, MaurizioFoijer, FlorisSchepisi, GiuseppeFont-Burgada, JoanSchildgen, OliverFranchina, TindaraScovassi, Anna IvanaFreeman, James WDi Sebastiano, PierluigiFribley, Andrew M.Seimiya, HiroyukiFrochot, CélineShackelford, Rodney E.Fujita, MitsuguShafirstein, GalFunel, NiccolaShah, NilayFurukawa, TatsuhikoSharafeldin, NohaFutakuchi, MitsuruShen, Jia ZackGao, YangShepherd, PeterGao, ShuaiSheth, SandeepGarcía-Jiménez, CustodiaShiao, Stephen L.Gatault, SolèneShim, HyunsukGe, XiaodongShinohara, ShogoGeorgakilas, AlexandrosShiozawa, YusukeGhigo, AlessandraShirato, HirokiGiardino, AlessandroShnyder, SteveGiuriato, SylvieSicard-Roselli, CécileGius, DavidSiegfried, JillGobe, Glenda C.Sihver, LembitGodar, Dianne E.Silva, Susana N.Gohda, EiichiSingh, AnilGolovleva, IrinaSingh, AnuragGonzalez, VictorSingh, Shree RamGonzález-González, RogelioSklar, Larry A.Grant, WilliamSkougaard, KristinGray, StevenSmahel, MichalGrivas, PetrosSmith, Robert A.Gross, GideonSoldevilla, MarioGrusch, MichaelSon, Deok-sooGuedan, SoniaSong, Jia L.Guha, Prajna P.Sonis, StephenGuillermet-Guibert, JulieSonnenberg, ArnoudGullberg, DonaldSottnik, JosephGuo, Zong ShengSprenger, CynthiaGustafsson, Jan-ÅkeSrivastava, AkhilHamanishi, JunzoSrivenugopal, KalkunteHamieh, MohamadStangl, HerbertHamilton, GerhardStefanini, LuciaHannafon, BethanySteinbrunn, TorstenHaque, InamulStewart, TenealeHarbin, Sherry L.Stickeler, ElmarHariharan, SeethalakshmiStockmann, ChristianHassan, MohamedStoker, Joshua B.Hata, YutakaStuber, MargaretHatzakis, NikosSu, RuiHeckman, CarolineSugii, YuhHellerbrand, ClausSugimoto, NaotoshiHeng, HenrySun, QianHildreth, BlakeSundar, ReshmaHiraoka, NobuyoshiSutcliffe, Julie L.Ho, YeungSuzuki, KeijiHofman, PaulSuzuki, RyusukeHojman, PernilleSwierczynski, JulianHolinstat, MichaelSzegezdi, EvaHolstein, SarahTaguchi, YoshihiroHori, ToshiyukiTaguchi, AyumuHsieh, James J.Takahashi, AkihisaHu, Dong GuiTakahashi, HirokiHung, Jan-jongTakeuchi, KengoHuppi, KonradTakiguchi, YuichiHusain, SirajTaniguchi, HiroyaHutchison, Robert E.Tanner, Julian A.Ignatius, Sai-HongTarallo, ValeriaIlmer, MatthiasTarasova, Nadya I.Inamura, KentaroTartarone, AlfredoInga, AlbertoTashima, ToshihikoIorio, MarilenaTatsuaki, KanaiGrand, Roger J.Testa, UgoJacobs, Aaron T.Thomas, SufiJacola, LisaTinguely, MarianneJames, McCaulTober, KathleenJang, Se JinTorigoe, ToshihikoJankovic, MomciloTrembley, Janeen H.Jardin, IsaacTremolada, MartaJasiulionis, Miriam G.Trøst Jørgensen, JanJean, DidierTschan, Mario P.Jeggo, PennyTsoulfas, GeorgeJenkins, Samir V.Turcotte, Lucie M.Jensen, Randy L.Turner, SuzanneJensen, Lars HenrikUhl, EberhardJeong, Keun-YeongVan Den Bergh, HubertJia, WilliamVan Den Brekel, Michiel Wilhelmus MariaJiang, Chen ChenVan Etten, JamieJiang, JianxiongVanderWeele, DavidJohansson, StaffanVassetzky, YegorJung, SeungyounVazquez, MarceloJung, KlausVega, FranciscoLauf, Peter K.Vella, Laura JayneKallis, YiannisVeltkamp, ChristianKallunki, TuulaVenturella, RobertaKanai, MasashiVerma, Navin KumarKang, HyunseokViggiano, DavideKashiwagi, ShinichiroVillalobo, AntonioKasinski, AndreaVincent, Vander PoortenKatiyar, Santosh K.Viola, GiampietroKawasumi, MasaokiVizeacoumar, FrancoKelly, JanetVoutsadakis, Ioannis A.Khan, ShafiqWahlestedt, ClaesKibriya, MuhammedWakimoto, HiroakiKim, Chang HyeukWang, XiangdongKim, Sang-WeWard, DouglasKim, JoongsunWatson, PhilipKim, Tae IlWedemeyer, HeinerKim, Sang-WeWeinberg, Robert A.Kim, JunghoonWeinhold, NielsKim, HyungjinWesterhout, Ellen M.Klampfer, LidijaWhelan, RebeccaKlapdor, RüdigerWhitelock, JohnKleeff, JörgWillard, Victoria W.Klokov, Dmitry Y.William, William NassibKobayashi, JunyaWilliams, Christopher C.Kohno, TakashiWinter, JörnKonishi, HirotakaWitkamp, Frederika EricaKornprat, PeterWu, LilyKosaka, NobuyoshiWu, XiaohuaKosinsky, Robyn LauraXie, QiKoul, HariXing, JianhuaKovacs, Pamela J.Xing, JunjiKrishna, Somashekar G.Xiong, ZhaohuiKrog Frandsen, StineXiong, Zheng-MeiKrug, SebastianXu, BaoshanKubo, ToshioXu, DazhongKurita, HirofumiYaddanapudi, KavithaKurozumi, KazuhikoYang, PeiKwong, JosephYang, Sien-SingKypta, RobertYao, JiayiLa Vecchia, CarloYeh, Shiou-HweiLachenmeier, DirkYip, Kay-pongLahooti, HooshangYokoyama, AkiraLai, Hsueh-ChouYu, Yan PingLai, RaymondYu, YanlinLanguino, Lucia R.Yuan, XueLathia, JustinYuan, ChunyanLaura, MansiYue, JunmingLaurenzana, AnnaZacksenhaus, EldadLeavenworth, JianmeiZadeh, GelarehLee, Kin Wah TerenceZamay, Anna S.Lee, Jung-MinZaravinos, ApostolosLee, Sang-HanZaret, Kenneth S.Leenstra, SiegerZhang, CaiguoLele, ShashikantZhang, WeijieLengerke, ClaudiaZhang, XiaodongLeong, HonZhou, LanLeong-Poi, HowardZhou, WenchaoLi, FengZi, XiaolinLi, CalvinZiegler, PatrickXiao, LifuZigrino, PaolaLin, KuiZu, YouliLin, Sue-HwaZuco, Valentina

